# AI-Driven Innovations for Quality Control and Standardization: Future Strategies in Adipose-Derived Stem Cell Manufacturing

**DOI:** 10.3390/ijms27052388

**Published:** 2026-03-04

**Authors:** Riccardo Foti, Gabriele Storti, Marco Palmesano, Alessio Calicchia, Roberta Foti, Guido Ciprandi, Giulio Cervelli, Maria Giovanna Scioli, Augusto Orlandi, Valerio Cervelli

**Affiliations:** 1 Plastic and Reconstructive Surgery, Department of Surgical Sciences, Tor Vergata University, 00133 Rome, Italy; riccardofoti.md@gmail.com (R.F.); marcopalmesano@gmail.com (M.P.); alessio.calicchia93@gmail.com (A.C.); valeriocervelli@virgilio.it (V.C.); 2 PhD Program in Applied Medical Surgical Sciences, Department of Surgical Sciences, University of Rome, 00133 Roma, Italy; 3 Division of Rheumatology, AOU Policlinico “G. Rodolico” San Marco, 95121 Catania, Italy; robertafoti@hotmail.com; 4 PhD programme Innovative Technologies in Biomedical Sciences, University of Enna “Kore”, 94100 Enna, Italy; 5 Plastic Reconstructive and Aesthetic Surgery Clinic, University Hospital of Padua, 35128 Padua, Italy; guidociprandi@gmail.com; 6 Department of Experimental Medicine, University of Rome “Tor Vergata”, 00133 Rome, Italy; giulio.cervelli@gmail.com; 7 Anatomic Pathology, Department of Biomedicine and Prevention, University of Rome Tor Vergata, 00133 Rome, Italy; scioli@med.uniroma2.it (M.G.S.); orlandi@uniroma2.it (A.O.)

**Keywords:** artificial intelligence, machine learning, deep learning, adipose-derived stem/stromal cells (ADSCs)

## Abstract

Artificial intelligence (AI), including machine learning (ML) and deep learning (DL), is increasingly transforming the study, manufacturing, and clinical translation of adipose-derived stem/stromal cells (ADSCs). ADSC-based therapies face persistent challenges related to donor variability, heterogeneous cell populations, limited standardization of culture protocols, and the need for robust quality control (QC) and potency assessment under Good Manufacturing Practice (GMP) conditions. This review discusses how AI-driven approaches can support the ADSC pipeline from donor and tissue pre-screening, through isolation and expansion, to differentiation and batch release decisions. We highlight major methodological advances in computer vision and label-free imaging for monitoring morphology, confluency, proliferation, senescence, and contamination, as well as AI-assisted optimization strategies for culture parameters and differentiation protocols. In addition, we examine the growing role of multi-omics integration (transcriptomics, proteomics, metabolomics, and secretomics) combined with ML to predict functional potency, stratify donors, and identify biomarkers associated with therapeutic efficacy. Finally, we address current limitations, including data scarcity, inter-laboratory variability, model interpretability, and regulatory requirements, and outline future perspectives such as closed-loop bioprocess control, foundation models, and federated learning frameworks. Overall, AI offers a powerful toolkit to improve the reproducibility, safety, and scalability of ADSC manufacturing and to accelerate the development of standardized, data-driven regenerative medicine products.

## 1. Introduction

Adipose-derived stem/stromal cells (ADSCs) are multipotent mesenchymal stromal cells obtained mainly from subcutaneous adipose tissue or lipoaspirates. They have strong proliferative capacity, multilineage differentiation potential, and a rich paracrine secretome. These properties, combined with minimally invasive harvesting and high cell yield, make ADSCs a powerful tool for regenerative medicine, immune modulation, and tissue engineering.

ADSCs have been described by their surface markers, their capacity to differentiate into several cell lineages, and their practical clinical advantages, including the fact that they can be obtained in much higher numbers than mesenchymal stem cells from bone marrow [1].

ADSCs also demonstrate potent regenerative and immunomodulatory effects, and their abundance, accessibility, and clinical versatility have been widely recognized. Indeed, Mazini et al. summarized the biological properties and therapeutic mechanisms of ADSCs in multiple clinical contexts [2].

Multiple pieces of evidence describe in detail how ADSCs act within the pathological environment, illustrating their contribution to angiogenesis, the attenuation of fibrosis, and the modulation of dysregulated immune pathways. These mechanisms—supported by paracrine secretion of growth factors, cytokines, and extracellular vesicles—are increasingly regarded as key elements that could be leveraged in clinical translation, particularly because they influence vascular repair, fibroblast activity, extracellular matrix remodeling, and the inflammatory milieu in a manner consistent with therapeutic goals [3,4,5,6,7,8,9,10,11].

ADSCs are increasingly recognized as a valuable resource in musculoskeletal and cartilage tissue engineering, where their behavior is strongly shaped by the surrounding microenvironment. Recent comparative research has shown that when these cells are cultured on different biomaterial scaffolds, their osteogenic and chondrogenic trajectories can shift markedly. Subtle variations in matrix composition, stiffness, and porosity are enough to guide ADSCs toward bone- or cartilage-like phenotypes, underscoring how responsive they are to biomechanical and biochemical cues. This sensitivity to scaffold design is now considered a central element in developing effective strategies for osteochondral repair, as it allows engineered constructs to better mimic native tissue architecture and functional demands [12,13,14].

Despite their promise, several factors continue to limit the standardization of ADSC-based therapies, reflecting challenges—ranging from manufacturing requirements to clinical protocol consistency and the complexity of cell behavior in different disease environments—that deserve careful consideration.

### 1.1. Factors Limiting the Consistent Clinical Use of ADSCs

#### 1.1.1. Heterogeneity of the Starting Material

Donor-related characteristics—including age, sex, body mass index (BMI), metabolic health, and systemic inflammation—significantly influence the biology and therapeutic potential of adipose-derived stem cells (ADSCs). Increasing donor age is associated with reduced ADSC proliferation, differentiation capacity, and altered paracrine activity, including decreased secretion of key growth factors such as VEGF and HGF, which may impair regenerative efficacy [15,16]. Sex differences can affect proliferation rates and cytokine responses, with some evidence of higher proliferative and inflammatory responses in female-derived ADSCs compared to male-derived cells [17].

Elevated BMI and metabolic disorders such as obesity and type 2 diabetes alter ADSC immunomodulatory properties, leading to increased expression of inflammatory markers, reduced immunosuppressive activity, and a shift toward a pro-inflammatory secretome. These changes compromise the ability of ADSCs to suppress lymphocyte proliferation and promote anti-inflammatory macrophage polarization, which is critical for tissue repair and immune regulation. Additionally, ADSCs from obese donors may exhibit reduced stemness and impaired migration and differentiation potential [18,19,20].

Systemic inflammation and donor health status further modulate ADSC phenotype and function, contributing to variability in cytokine and growth factor secretion profiles [21]. This intrinsic heterogeneity complicates the establishment of universal processing protocols and reliable quality-control standards for ADSC-based therapies, as highlighted by the lack of standardized immunopotency assays.

#### 1.1.2. Variability in Culture Conditions

Differences in serum/xeno-free media, oxygen tension, passage number, and scaffold architecture, as shown by Fiorelli et al., profoundly alter ADSC behavior, affecting both experimental reproducibility and clinical potency [12].

#### 1.1.3. Senescence and Loss of Function

During in vitro expansion, ADSCs progressively enter replicative senescence, a process that diminishes their regenerative performance and promotes the emergence of a pro-inflammatory secretory profile (SASP). Increasing evidence has clarified the molecular pathways driving this transition and highlighted how senescence may influence safety, efficacy, and reproducibility in clinical applications [16].

#### 1.1.4. Quality Control and Phenotypic Characterization

Given the substantial donor-to-donor variability in the secretome and antifibrotic activity of ADSCs, and the limited predictive value of standard MSC markers (CD73, CD90, CD105), the current approaches for improved, functional quality control of ADSCs focus on functional potency assays and advanced molecular profiling.

Functional potency assays, such as immunomodulatory activity (e.g., T-lymphocyte proliferation inhibition), differentiation capacity, and antifibrotic effects, are increasingly recommended to assess the therapeutic potential of ADSCs beyond surface marker expression. These assays directly measure the biological activity relevant to the intended clinical application and are considered critical quality attributes (CQAs) for cell product release [22]. Single-cell transcriptomic and proteomic profiling is emerging as a powerful tool to characterize ADSC heterogeneity and predict functional outcomes. High-resolution molecular analyses can identify subpopulations with enhanced regenerative or immunomodulatory properties, enabling more precise selection and enrichment of therapeutically potent cells. For example, the identification of ALP+/CD73+ subpopulations correlates with enhanced osteogenic potential and may serve as a functional marker for cell selection [9,23,24]. Standardization of manufacturing processes, including defined processing quantitative and qualitative standards (PQSs and PQLSs), is also recommended to minimize variability and ensure reproducibility in cell isolation and expansion [25]. Additionally, assessments of senescence markers, genetic stability, and cell viability are integral to quality control frameworks [11].

#### 1.1.5. Lack of Standardized Expansion Protocols

Variations in isolation methods and culture conditions for ADSCs directly affect cell potency and functional profiles. Enzymatic digestion and mechanical isolation yield populations with differing compositions, viability, and differentiation potential: mechanical approaches often preserve viability and immunomodulatory capacity but produce lower yields, whereas enzymatic methods increase progenitor diversity yet may alter surface markers and function [26,27]. Device choice, processing parameters, and reagent variability further influence cellularity and secretome characteristics [28,29].

Culture conditions—including medium formulation, oxygen levels, and passage number—also shape ADSC proliferation, differentiation, and paracrine activity. Xenogeneic-free systems can reduce immunogenic and contamination risks but may modify growth dynamics and secretory patterns [30]. Even when protocols are aligned, inter-laboratory variability and donor-specific differences remain significant [31].

These inconsistencies complicate the definition of standardized products and hinder regulatory approval. The absence of universally accepted processing standards and CQAs (critical quality attributes) limits reproducibility, cross-study comparability, and safety evaluation [32]. Although standardization efforts are ongoing, current methodological heterogeneity continues to impede clinical translation and regulatory acceptance of ADSC-based therapies [33].

#### 1.1.6. AI, ML, and DL as Solutions to These Challenges

Artificial intelligence (AI), including machine learning (ML) and deep learning (DL), is emerging as a transformative tool to address the limitations above.

DL has already revolutionized cell-image analysis, improving segmentation, morphology quantification, and dynamic tracking. Indeed, a landmark overview by Moen et al. summarizes applications of DL in cellular imaging and demonstrates its capacity for automated phenotype characterization [34]. Moreover, ML methods likewise enable integration of high-dimensional omics data, supporting donor classification, predictive potency modeling, and optimization of culture protocols.

In addition, a foundational review by Angermueller et al. highlighted how ML-based multimodal data integration can decode lineage trajectories and biological states—concepts highly relevant to ADSC research [35].

**Figure 1:**
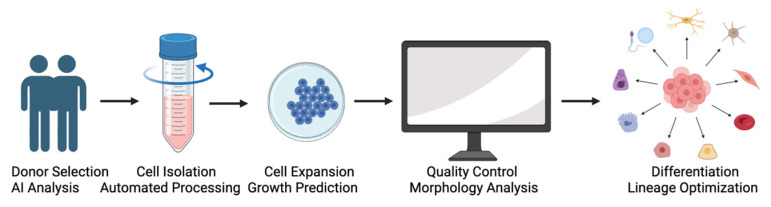
AI-enabled workflow for ADSC manufacturing and differentiation. Schematic overview of the adipose-derived stem/stromal cell (ADSC) pipeline, highlighting key stages where artificial intelligence (AI) can support standardization and decision-making. AI tools can assist in donor selection, automate isolation procedures, predict expansion and growth kinetics, enable image-based quality control through morphology analysis, and optimize differentiation protocols toward specific lineages.

Together, these developments establish the rationale for applying AI across the ADSC pipeline—from donor selection and culture monitoring to differentiation optimization and GMP manufacturing. Although several reviews have explored artificial intelligence applications in stem-cell research or discussed the biological properties of adipose-derived stem/stromal cells (ADSCs), few have systematically integrated AI methodologies within a Good Manufacturing Practice (GMP)–oriented quality-control framework specific to ADSC manufacturing. In particular, prior literature has generally addressed imaging analytics, multi-omics integration, or differentiation modeling in isolation, without mapping these approaches across the entire ADSC pipeline—from donor selection to batch release decision-making. The present review uniquely addresses this gap by providing an end-to-end, GMP-aligned framework that connects AI-driven data analytics to critical quality attributes (CQAs), potency assessment, and regulatory decision-support in ADSC biomanufacturing. An overview of AI applications across the entire ADSC workflow—from donor selection to GMP bioprocessing—is provided in Table 1. As summarized in Figure 1, AI-based tools can support multiple steps of the ADSC manufacturing pipeline, from donor selection to differentiation optimization.

#### 1.1.7. Search Strategy and Scope

This manuscript was conceived as a narrative review aimed at providing a comprehensive and forward-looking overview of artificial intelligence (AI) applications in adipose-derived stem/stromal cell (ADSC) research and manufacturing. Although not designed as a systematic review, a structured literature search was conducted to ensure broad coverage and methodological transparency.

Relevant studies were identified through searches in PubMed, Scopus, and Web of Science. The primary search window covered publications from 2010 to 2025, with particular focus on studies published after 2015, reflecting the rapid expansion of AI-driven methodologies in biomedical research. Combinations of keywords included: “adipose-derived stem cells” OR “ADSC”, “mesenchymal stromal cells” OR “MSC”, combined with “artificial intelligence”, “machine learning”, “deep learning”, “computer vision”, “bioprocessing”, “quality control”, “multi-omics”, and “differentiation”.

Inclusion criteria comprised original research articles, methodological studies, and high-quality reviews describing AI-based approaches applicable to stem-cell biology, biomanufacturing, culture monitoring, differentiation analysis, or potency prediction. Studies were prioritized when directly involving ADSCs. However, given the limited number of ADSC-specific AI studies currently available, selected examples from closely related mesenchymal stromal cell (MSC) systems or other stem-cell platforms were included when the AI methodology was transferable to ADSC isolation, expansion, differentiation, or GMP-oriented manufacturing workflows.

Exclusion criteria included studies lacking methodological clarity, non-peer-reviewed reports, and AI applications unrelated to cell biology or manufacturing.

This structured yet narrative approach was adopted to balance methodological transparency with the exploratory and integrative nature of the review, while explicitly acknowledging the evolving state of AI applications in ADSC research.

## 2. Overview of Artificial Intelligence Techniques

Artificial intelligence platforms integrate label-free imaging and sensor-derived data to quantitatively monitor ADSC morphology, proliferation, differentiation status, and culture stability, enabling objective tracking of critical quality attributes (CQAs) [36,37]. In ADSC cultures, where donor variability, stromal vascular fraction heterogeneity, and passage-dependent senescence introduce significant biological variability, automated feature extraction reduces subjectivity and improves reproducibility compared to manual assessment [38].

For ADSCs, multimodal integration is particularly relevant because functional potency often correlates imperfectly with standard MSC surface markers, requiring combined morphological, metabolic, and molecular readouts to define reliable release criteria [39]. Automated feedback systems can adjust culture conditions (e.g., media composition, oxygen tension) to optimize cell health and potency, reducing operator variability and improving reproducibility [40].

In summary, AI enables automated, scalable, and reproducible monitoring and optimization of ADSC cultures by providing real-time, quantitative assessment of cell morphology and functional status, supporting robust quality control and process standardization for clinical and manufacturing applications. Key AI-related terms, as well as the main AI techniques, required data inputs, ADSC-specific applications, and current limitations are summarized in Table 2 and Table 3.

In ADSC systems, regression and ensemble models are particularly useful for integrating donor metadata and omics features to classify cell states and predict functional outcomes in stem cell systems, particularly to address ADSC variability and quality control.

Supervised models—including regression, ensemble methods, and support vector machines—have been applied to classify ADSC subpopulations and predict functional outputs from high-dimensional datasets such as scRNA-seq and imaging features [41,42]. In ADSCs, these approaches are particularly useful for modeling donor-dependent differences in proliferation kinetics, immunomodulatory potency, and lineage bias, which are not captured by canonical MSC marker panels alone [43].

Unsupervised clustering helps resolve transcriptionally and morphologically distinct ADSC subpopulations, supporting identification of lineage-primed or senescence-prone subsets within nominally homogeneous cultures [44]. Deep learning models, including convolutional neural networks (CNNs), further enhance functional prediction by analyzing cell morphology and imaging data, allowing non-invasive, high-throughput assessment of differentiation status and multipotency [45].

Integrating these machine learning approaches with single-cell omics and imaging data enables precise classification of ADSC subpopulations, prediction of functional outcomes, and identification of critical quality attributes, thereby supporting improved quality control and standardization in stem cell biomanufacturing [46].

For example, regression- and tree-based models have proven effective in analyzing high-dimensional biological data and identifying relevant predictive features [47,48]. Furthermore, unsupervised clustering approaches remain essential tools for the identification of heterogeneous subpopulations in cell cultures and for stratifying donors based on cell behavior [49].

CNNs are widely used for microscopy-based segmentation and morphology quantification, and are particularly relevant for detecting subtle ADSC morphological changes during expansion and senescence [34,50,51]. Moreover, CNN-based segmentation and detection methods now achieve high accuracy in label-free analyses and support continuous monitoring of stem cell health and morphology [52].

Finally, predictive modeling and optimization algorithms such as Bayesian optimization, reinforcement learning, and evolutionary strategies offer powerful tools for tuning culture parameters [35].

### Model Generalizability, Dataset Migration and External Validation

While the technical performance of ML and DL models is increasingly demonstrated in controlled experimental settings, their versatility across different laboratories, donor populations, and clinical production environments remains a critical translational challenge. In the context of ADSC manufacturing, models trained on single-center datasets may be affected by domain shift when applied to external cohorts. Differences in imaging hardware, culture media formulations, passage number, oxygen tension, operator handling, and sensor calibration can significantly alter data distributions, leading to performance degradation when models are deployed outside their original training environment. Dataset migration therefore represents a central issue for AI implementation in ADSC workflows. Robust deployment requires external validation on independent datasets generated in different laboratories and under heterogeneous GMP-compliant conditions. Prospective validation studies and multi-center data integration are essential to ensure model stability and reproducibility. Approaches such as domain adaptation, transfer learning, federated learning frameworks, and standardized data preprocessing pipelines may mitigate cross-site variability and improve generalizability. In particular, federated learning strategies could allow collaborative model training across GMP facilities without direct sharing of sensitive donor-level data, thereby preserving privacy while increasing dataset diversity. 

From a practical perspective, classical machine-learning models such as random forests and support vector machines are generally more suitable for small-to-medium ADSC datasets and offer higher interpretability, especially when combined with feature-importance analysis. In contrast, deep-learning models such as convolutional neural networks typically require larger annotated datasets but provide superior performance in high-dimensional image-based tasks. Bayesian optimization and reinforcement learning are primarily process-optimization tools and can operate efficiently even with limited experimental iterations, making them attractive for culture-parameter tuning in GMP settings. Thus, model selection in ADSC research should balance interpretability, dataset size, and regulatory constraints rather than relying solely on predictive accuracy.

## 3. AI for ADSC Isolation and Yield Prediction

AI can be applied during the isolation step to reduce donor-to-donor variability in adipose-derived stem cell yield and quality by using predictive modeling based on donor characteristics such as age, sex, adiposity, and metabolic status. Machine learning algorithms—including regression models, random forests, and support vector machines—can be trained on large datasets that link donor variables to measured ADSC yield, viability, and functional markers. These models can then predict expected cell yield and quality from new donors prior to tissue processing, enabling selection of optimal donors or adjustment of isolation protocols to compensate for anticipated variability [53,54,55]. For instance, in mesenchymal stromal cells, random-forest models trained on data from more than 170 donors have been used to predict passage-specific population doubling times from variables including donor age and culture conditions, demonstrating that machine learning can capture how donor features influence expansion potential and growth kinetics [56]. Likewise, deep-learning–based analysis of histological slides has been used to quantify the proportions of adipose, stromal, and epithelial compartments in benign breast tissue in relation to anthropometric variables across life, supporting the concept that body size and composition are systematically linked to tissue architecture in a way that AI can detect and model [57]. By analogy, similar models could be trained to predict ADSC yield or viability from routine clinical variables (age, BMI, metabolic syndrome, etc.) combined with imaging-derived tissue features.

A second area of development is the use of ML- and Bayesian-optimization frameworks to tune isolation and processing parameters, such as enzymatic digestion time, enzyme concentration, and centrifugation regimes. In bioprocessing and cellular agriculture, multi–multi-information-source Bayesian optimization has been applied to optimize a 14-component culture medium for C2C12 cells, achieving more than a 180% increase in cell number compared with standard medium while requiring substantially fewer experiments than classical design-of-experiments approaches [58]. Robotic AI platforms combining laboratory automation with batch Bayesian optimization have also been used to search high-dimensional parameter spaces for stem-cell differentiation protocols, improving induced pluripotent stem cell–derived retinal pigment epithelial cell production by 88% relative to a pre-optimized protocol while exploring only a tiny fraction of the possible combinations [59]. Translating such strategies to ADSC isolation would allow data-driven optimization of digestion protocols and washing/centrifugation sequences to maximize viable stromal vascular fraction and ADSC recovery.

Finally, deep learning applied to histological and imaging data from adipose tissue can provide objective measures of tissue quality before isolation. Three-dimensional light-sheet microscopy combined with Mask R-CNN–based segmentation has been used to quantify crown-like structures and macrophage infiltration in intact adipose tissue, revealing distinct structural subpopulations and obesity-associated changes that serve as histological biomarkers of tissue inflammation [60]. At the organ level, radiomic profiling of perivascular adipose tissue with machine-learning classifiers has been shown to capture inflammation, fibrosis, and microvascular remodeling and to improve prediction of major adverse cardiac events over standard risk factors [61].

These examples show that AI can extract quantitative descriptors of adipose tissue inflammation, fibrosis, and microarchitecture; in an ADSC workflow, similar DL pipelines could be used to pre-screen lipoaspirate or adipose biopsies, flagging samples with unfavorable inflammatory or fibrotic profiles and thus supporting more consistent ADSC yield and potency.

## 4. AI for Monitoring Cell Culture

AI enables real-time, automated, and quantitative monitoring of cell cultures, reducing operator-dependent variability and supporting standardized workflows for ADSC expansion. Through deep learning, machine learning, and computer vision, it is now possible to track morphology, proliferation, senescence, contamination, and overall culture quality using label-free imaging, which is highly attractive for clinical-grade ADSC manufacturing.

### 4.1. Computer Vision for Cell Morphology

CNNs and other deep learning architectures have been widely applied to quantify basic morphological parameters in cell culture, such as confluency, cell shape, and cell density. For instance, Yi and colleagues developed a deep fully convolutional network based on U-Net to perform high-throughput, label-free cell detection and counting directly from diffraction patterns acquired by digital holographic microscopy, achieving ~99% accuracy in cell counting and demonstrating the feasibility of automated confluency estimation from holographic data [62].

More specialized pipelines have been proposed for MSCs, which are closely related to ADSCs. Indeed, Mota et al. developed an automated segmentation and ML-based phenotype classification framework using phase-contrast images of MSCs; after segmenting individual cells, morphometric and textural features were used to train classifiers that distinguished different MSC phenotypes with high accuracy, illustrating how morphology-based ML can support objective culture assessment [63].

Beyond simple morphology, ML models have been used to infer differentiation status from label-free images. For example, Kong et al. built a machine-learning pipeline that combines quantitative imaging from a label-free microscopy system with feature-based classifiers to discriminate undifferentiated and differentiated MSCs, providing an automated readout of differentiation state without fluorescent markers [44]. Similar morphology-based approaches could be adapted to predict ADSC differentiation into adipogenic, osteogenic, or chondrogenic lineages directly from phase-contrast or bright-field images.

AI has also been applied to the detection of senescent cells, a critical issue for stem-cell therapies because senescence impairs regenerative potential. Recently, Kusumoto et al. developed Deep-SeSMo, a morphology-based CNN system that identifies senescent cells from phase-contrast images and generates a quantitative “senescence score” used to screen for anti-senescent drugs [64]. Moreover, Duran et al. showed that senescence can be detected robustly using ML algorithms trained on nuclear morphology features extracted from high-content imaging, providing an additional non-invasive route for senescence monitoring [65]. Both strategies are directly relevant to ADSC cultures, where early identification of senescent subpopulations is essential for maintaining therapeutic potency.

### 4.2. Non-Invasive Culture Monitoring

A central advantage of AI-driven imaging is the ability to monitor cultures non-invasively over time. Indeed, time-lapse phase-contrast and bright-field imaging, combined with DL, allow quantification of cell motility, proliferation, and morphological dynamics without the need for labels. Jang and colleagues developed MARS-Net, a deep learning-based segmentation pipeline that accurately localizes cell edges across multiple live-cell microscopy modalities, enabling robust quantification of cellular morphodynamics in long-term time-lapse datasets [66]. Such segmentation frameworks can be applied to ADSC cultures to measure proliferation rates, migration, and colony behavior during expansion.

Self-supervised DL approaches have further improved dynamic culture monitoring. Wu et al. introduced DynaMorph, a self-supervised model that learns quantitative “morphodynamic” representations from live-cell imaging, enabling data-driven definition of cell states and trajectories based solely on time-lapse morphology [67]. Similarly, Copperman et al. used deep learning to embed single-cell morphodynamic trajectories from live imaging into a low-dimensional landscape, providing a powerful framework to study cell-state transitions and drug responses in vitro [68]. These approaches can, in principle, be trained on ADSC time-lapse data to infer growth rates, optimal passage timing, and early deviations from healthy culture trajectories.

Non-invasive AI analysis has also been used to predict molecular markers and functional states directly from label-free images. Akiyoshi et al. developed an ML-based method that predicts the expression of pluripotency markers (e.g., OCT4, NANOG) and other multimodal molecular readouts in induced pluripotent stem cells from bright-field images alone, providing a template for similar non-destructive assessment of ADSC potency markers [69]. Complementing this, Asmar et al. described an AI pipeline for high-volume, label-free imaging of iPSC colonies that automatically performs nuclear segmentation and mitosis detection from phase-contrast images, demonstrating how DL can monitor proliferation and mitotic activity at single-cell resolution in large-scale cultures [70]. These non-invasive strategies are directly transferable to ADSC expansion, where destructive sampling should be minimized.

### 4.3. Image-Based Quality Control

Image-based AI pipelines are increasingly recognized as a cornerstone for quality control (QC) in stem-cell manufacturing. Indeed, label-free optical imaging modalities (e.g., multiphoton metabolic imaging, hyperspectral imaging, Raman spectroscopy, phase-contrast) can be combined with ML algorithms to rapidly assess stem-cell viability, differentiation state, and metabolic status throughout the manufacturing workflow, emphasizing their potential for integrating automated QC into bioprocess pipelines [71].

Several concrete examples illustrate how DL can distinguish desired stem cells from unwanted cell types or states on the basis of morphology alone. For instance, Kong et al. demonstrated that label-free imaging combined with ML accurately classifies MSCs at different stages of osteogenic differentiation, providing an automated readout of differentiation status [44]. A recently developed deep-learning approach, Bright2Nuc, enables the prediction of nuclear fluorescence in 3D pluripotent stem-cell cultures directly from bright-field images. This virtual-staining strategy allows label-free evaluation of cell density, nuclear morphology, and overall structural organization within microfluidic systems [71]. Virtual-staining strategies like this could similarly be used to monitor ADSC nuclei, chromatin organization, or cell-cycle status in real time.

AI-driven QC also extends to the detection of senescent or damaged cells within therapeutic cell batches. As discussed above, morphology-based CNN systems such as Deep-SeSMo identify senescent cells and quantify their prevalence in culture [64]. In parallel, Duran et al. used nuclear morphology and ML to detect senescence across multiple tissues and conditions, providing a generalizable framework for senescence detection that could be applied to ADSC manufacturing [65]. These tools could be integrated into automated imaging platforms for ADSCs to ensure that senescent or otherwise functionally compromised cells are kept below predefined thresholds.

Finally, several recent reviews highlight the broader potential of DL-enhanced live-cell imaging for robust, real-time QC in complex cell culture systems. For instance, Pylvänäinen et al. discussed how DL methods for segmentation, tracking, denoising, and artifact correction are transforming live-cell microscopy into a quantitative, high-throughput measurement tool, with clear implications for industrial stem-cell bioprocessing [72]. Together, these developments indicate that AI-based, image-driven QC pipelines can provide continuous, non-invasive surveillance of ADSC cultures, supporting safer and more consistent cell-therapy products.

### 4.4. Domain Shift and Robustness in Imaging-Based AI

A critical yet often underappreciated limitation of imaging-based AI models in stem-cell manufacturing is their sensitivity to domain shift. Domain shift refers to discrepancies between the data distribution used during model training and the data encountered during deployment. In ADSC culture monitoring, such discrepancies may arise from differences in microscope hardware, illumination settings, camera sensors, magnification levels, culture vessel types, confluency extremes, presence of debris or air bubbles, focus drift, or laboratory-specific imaging protocols.

Even high-performing convolutional neural networks (CNNs) trained under controlled conditions may exhibit substantial performance degradation when applied to images acquired in different laboratories or under altered acquisition parameters. In GMP-oriented settings, where reproducibility and cross-site consistency are essential, such brittleness represents a significant translational barrier.

To mitigate these risks, several strategies may be employed. These include multi-site training datasets to increase model generalizability; data augmentation techniques simulating realistic variations in brightness, contrast, blur, and noise; domain adaptation and transfer-learning approaches; and explicit out-of-distribution (OOD) detection systems capable of flagging images that deviate significantly from the training domain. Additionally, continuous performance monitoring and re-validation procedures should be implemented whenever hardware, imaging protocols, or culture conditions are modified.

Incorporating domain shift detection and robustness evaluation into AI validation workflows is particularly important when image-based models contribute to quality control or batch release decisions. Addressing this challenge is therefore essential for translating AI-driven microscopy tools from experimental settings to standardized, multi-center ADSC manufacturing environments.

## 5. AI for Optimizing Culture Conditions

AI-driven modeling is increasingly used to rationally design and control culture environments, moving beyond trial-and-error protocols and toward quantitative, data-driven optimization of media composition, feeding strategies, and process parameters. Although most published work focuses on mammalian cell lines or immune cells rather than ADSCs specifically, the same methodological frameworks can be translated to ADSC expansion and differentiation.

### 5.1. Machine-Learning Models for Media Composition

Several research groups have shown that supervised and active-learning approaches can efficiently optimize complex, multi-component media. Indeed, Hashizume and Ying reviewed how high-throughput experimentation coupled with algorithms such as random forests, support vector machines, and Bayesian optimization can identify non-intuitive combinations of nutrients and supplements that maximize cell growth and productivity while reducing experimental burden [73].

Building on this concept, the same authors recently also developed a “biology-aware” ML platform that integrates error-aware preprocessing, predictive modeling, and active learning to iteratively refine a 57-component serum-free medium for CHO-K1 cells, achieving ~60% higher peak cell concentration than commercial media with only 364 tested formulations [74].

A complementary Bayesian-optimization framework was applied to human peripheral blood mononuclear cells (PBMCs) and Komagataella phaffii cultures, where iterative modeling of experimental data identified cytokine cocktails and carbon-source mixtures that improved viability or recombinant protein productivity using 3–30-fold fewer experiments than traditional design-of-experiments strategies [75].

In the end, these studies demonstrate that ML-guided optimization can efficiently search high-dimensional media spaces, a strategy that could be directly adapted to ADSC culture to tune growth factors, serum alternatives, and small-molecule supplements for maximal expansion and maintenance of stemness.

### 5.2. Reinforcement Learning for Dynamic Control of Culture Conditions

Reinforcement learning (RL) offers a framework to actively control culture conditions—such as feeding rate, dissolved oxygen, and temperature—based on continuous feedback from the system. Recent work has applied deep reinforcement learning to the regulation of microbial co-cultures in both simulated and experimental bioreactors, enabling control agents to learn optimal strategies for stabilizing species ratios and enhancing productivity by dynamically adjusting feed composition in real time [76].

Although this work was performed on microbial systems, it illustrates how RL can integrate sensor data (e.g., optical density, metabolite levels, or online imaging) to continuously adjust process parameters. The same paradigm could be applied to ADSC bioreactors, where an RL controller would tune oxygen tension, perfusion rate, or feeding schedules to maintain desired growth trajectories and prevent over-confluence or nutrient depletion, especially when combined with ML-optimized media recipes [73,75].

More generally, approaches that integrate mechanistic metabolic models with data-driven methods are being explored to refine fermentation parameters and operational strategies, reinforcing the notion that AI-driven controllers can be incorporated into modern bioprocesses to achieve more robust and adaptive culture management [77].

### 5.3. Predictive Modeling for Expansion Kinetics and Batch-to-Batch Reproducibility

Predictive models that link early, non-invasive measurements to later expansion performance are crucial for ensuring batch reproducibility in ADSC manufacturing. Recent advances have shown that morphology-based machine-learning models, trained on time-lapse phase-contrast images, can predict both growth rate and T-cell proliferation–inhibitory potency in mixed bone-marrow and adipose-derived MSC cultures. Such models are able to flag donor lots with unfavorable profiles with very high accuracy (above 0.95) and to forecast expansion kinetics using only the first few days of morphological data, illustrating how early image-derived features can reliably anticipate later growth behavior and immunomodulatory performance [78].

Moreover, complementary work by Mota et al. combined automated MSC segmentation with machine-learning-based phenotype classification, using morphometric and textural features from phase-contrast images to discriminate between desirable and less efficacious MSC phenotypes during expansion [63]. The pipeline achieved high sensitivity and specificity for both cell detection and phenotype classification, enabling standardized, image-based assessment of culture quality over time.

Finally, Klontzas and colleagues integrated metabolomics with gradient-boosting ML models to predict osteogenic differentiation of mesenchymal stem cells in 2D and 3D cultures, identifying a small panel of metabolites whose levels strongly correlated with mineralization and served as quantitative potency markers for advanced therapy medicinal products [79].

When combined, these studies demonstrate how AI can link early morphological or metabolic readouts to later expansion and differentiation outcomes. Applied to ADSCs, similar ML pipelines could be trained on donor metadata, imaging, and omics features to predict culture growth curves, optimal passage windows, and final product potency, thereby improving batch-to-batch reproducibility in clinical-grade manufacturing.

## 6. AI in Differentiation Protocol Optimization

AI is increasingly used to optimize differentiation protocols for ADSCs, improving control over lineage commitment and maturation toward adipogenic, osteogenic, chondrogenic, and neural phenotypes. By integrating imaging, multi-omics, and time-lapse culture data, ML and deep learning (DL) models can quantify differentiation kinetics, identify key signaling pathways, and predict lineage potential from baseline molecular profiles [80,81]. Recent omics-focused reviews emphasize that integrating large-scale datasets from ADSCs will require advanced AI tools to decode epigenetic and transcriptional signatures that govern lineage choice and differentiation robustness [82].

### 6.1. AI for Adipogenic Differentiation

Adipogenic differentiation of ADSCs is one of the best-studied use cases for AI-based optimization. A deep learning–based live-cell imaging system has been used to quantify adipogenic differentiation kinetics of human ADSCs by tracking adipose area and lipid droplet formation over time. Using CNNs on phase-contrast images, the method captured donor-specific kinetics and allowed quantitative analysis of how culture conditions affect adipogenesis, suggesting that similar DL pipelines could be used to systematically optimize induction protocols (e.g., growth factor combinations or induction timing) [83].

Complementary work has applied ML to vibrational spectroscopy readouts during adipogenesis. In human adipose-derived mesenchymal stem cells (AD-MSCs), Fourier-transform infrared (FTIR) and Raman spectra recorded over the course of adipogenic induction were analyzed with supervised ML to classify differentiation stages and identify spectral markers associated with lipid accumulation and membrane remodeling, providing non-invasive readouts that could be used to fine-tune media composition and timing in adipogenic protocols [84].

From a metabolic perspective, AI has also been used to monitor ADSC differentiation in a label-free manner. In adipose-tissue-derived human mesenchymal stem cells undergoing adipogenic and osteogenic differentiation, ^1H–^1H TOCSY 2D NMR spectra were analyzed using kernel-based ML classifiers to identify metabolic fingerprints associated with each differentiation trajectory. This approach demonstrated that ML can automatically detect subtle metabolic shifts during lineage commitment, supporting predictive modeling of differentiation outcome and providing a framework for rational adjustment of induction conditions [85].

### 6.2. AI for Osteogenic and Chondrogenic Differentiation

For osteogenic differentiation, single-cell and multi-omics datasets provide rich input for AI models that aim to predict osteogenic potential and identify regulatory pathways. A single-cell RNA-seq and CellTagging study on human ADSCs reconstructed clonal differentiation trajectories and identified a subpopulation with strong osteogenic predisposition. By combining lineage tracing and transcriptomics, the authors pinpointed genes such as SERPINE2, SFRP1, KRT7, PI16, and CPE as key regulators of successful osteogenic induction, illustrating how feature-importance analysis on high-dimensional transcriptomic data can reveal molecular determinants of osteogenic differentiation and guide optimization of osteoinductive cocktails [80].

Moreover, recent integrative omics analyses of ADSCs highlight how transcriptomic, proteomic, and lipidomic differences between donors and culture conditions shape their osteogenic and chondrogenic potential. These reviews explicitly point out that applying ML and AI to multi-omics datasets will be essential to define predictive signatures of lineage commitment and to systematically optimize differentiation protocols (e.g., growth factor combinations, mechanical cues, and timing) for bone and cartilage applications [82].

Although explicit chondrogenic ADSC studies with DL/ML are still limited, the same conceptual framework—multi-omics feature selection, trajectory inference, and image-based classifiers—can be directly transferred to chondrogenic protocols. Integrating gene-expression trajectories, extracellular matrix composition, and imaging of cartilage-like matrix deposition is a natural next step for AI-driven optimization of ADSC-derived chondrocytes, as suggested by these multi-omics analyses.

### 6.3. AI for Neural Differentiation of ADSCs

Neural differentiation of ADSCs is another area where AI and advanced analytics are emerging as key tools. Recent research used integrative single-cell RNA-seq and ATAC-seq to map the evolutionary trajectory of ADSCs induced into astrocytes. By applying pseudotime and trajectory inference, together with motif and footprint analysis, the study identified NFIA/B/C/X and CEBPA/B/D as critical transcription factors orchestrating astrocytic differentiation and showed that chromatin accessibility changes precede transcriptional activation. Although the analysis did not rely on classic “black-box” DL, it uses computational methods similar to ML for high-dimensional feature extraction and trajectory modeling, providing a blueprint for how AI could be used to rationally adjust timing and composition of neural induction protocols [86].

Additionally, single-cell RNA-seq has been used to describe the “stemness landscape” of mature ADSCs, identifying transcriptional clusters with high or low stemness scores and mapping their pseudo-temporal position along growth and differentiation trajectories. Such datasets are ideal input for ML models that predict differentiation potential (e.g., toward neural vs. mesodermal lineages) from baseline transcriptomic profiles, enabling pre-selection of ADSC subpopulations best suited for neural differentiation [87].

### 6.4. AI Use Cases Across Lineages

#### 6.4.1. Identifying Key Signaling Pathways via Feature Importance

Across adipogenic, osteogenic, and neural differentiation, AI models—especially those trained on single-cell and multi-omics datasets—can use feature importance analysis (e.g., gradient-based importance, SHAP values) to highlight transcription factors, signaling pathways, and metabolic nodes that drive successful differentiation. Studies on osteogenic ADSC differentiation with CellTagging and global omics analyses of ADSCs already demonstrate how gene-level features such as SERPINE2, SFRP1, or lineage-biased miRNAs can emerge as key regulators; these can then be targeted experimentally by adjusting growth factors or small-molecule modulators in differentiation protocols [80,82].

#### 6.4.2. Predicting Differentiation Potential from Baseline Transcriptomics

Single-cell RNA-seq analyses of ADSCs show that even “undifferentiated” cultures contain transcriptionally distinct subpopulations with different stemness and lineage biases. By training ML classifiers or regression models on such data, it becomes feasible to predict the likelihood that a given ADSC subset will successfully undergo adipogenic, osteogenic, chondrogenic, or neural differentiation—information that can guide donor selection, pre-sorting, or customized induction protocols [80,87].

#### 6.4.3. DL for Analyzing Immunofluorescence and Morphology Patterns

Although most ADSC-specific work has focused on phase-contrast or bright-field images, there is increasing evidence from broader MSC research that CNN-based analysis of immunofluorescence and morphology profiles can predict functional properties such as immunosuppressive potency and growth rate. For example, a morphology-based ML pipeline using time-course phase-contrast images of bone marrow– and adipose-derived MSCs accurately predicted T-cell proliferation inhibitory potency and growth rate from non-invasive image features, highlighting the power of image-based models for functional phenotyping [78]. By analogy, similar DL architectures can be trained on ADSC immunofluorescence panels (e.g., lineage markers, transcription factors) and morphological features to classify differentiation stages and evaluate protocol performance in a label-efficient, high-throughput manner.

Together, these studies show that while AI-driven optimization of ADSC differentiation protocols is still emerging, there is already a solid foundation—especially in adipogenic and osteogenic systems, and increasingly in neural differentiation—on which more systematic, ADSC-specific ML/DL optimization frameworks can be built. Representative studies applying AI to ADSCs (or closely related MSC systems) are summarized in Table 4.

## 7. Multi-Omics and AI Integration

The integration of multi-omics datasets with AI is transforming the way ADSCs are characterized, classified, and selected for therapeutic applications. By combining transcriptomics, proteomics, metabolomics, and secretomics with machine-learning models, it is now possible to predict therapeutic potency, identify biomarkers associated with high-quality ADSC preparations, and stratify donors according to regenerative potential.

### 7.1. Transcriptomics + AI

Transcriptomic profiling of ADSCs has dramatically expanded in the past decade, offering large datasets ideally suited for ML-based feature selection and donor classification. Single-cell transcriptomic studies, in particular, reveal considerable heterogeneity within ADSC populations.

A recent study used single-cell RNA-seq to map the “stemness landscape” of adult human ADSCs and identify transcriptionally distinct subpopulations with variable differentiation potential and immunomodulatory profiles. Although no ML classifier was trained in this study, the authors explicitly highlight that these gene-expression clusters can be used to develop predictive ML models for donor stratification and potency assessment [87]. Similarly, Feng and colleagues reviewed transcriptomic and epigenomic datasets from ADSCs—including DNA methylation, histone modifications, and non-coding RNAs—and emphasized the central role of integrating multi-omics and ML approaches to predict ADSC lineage preferences, regenerative performance, and donor-specific therapeutic responses [82].

These transcriptomic datasets provide the foundation for developing ML models that can rank donors, identify high-stemness clusters, and detect transcriptional biomarkers predicting therapeutic potency.

### 7.2. Proteomics + ML

Proteomic profiling offers a quantitative understanding of ADSC functionality, revealing proteins linked to differentiation, inflammation, angiogenesis, and immunomodulation. Although ML applications in ADSC proteomics are still emerging, high-throughput proteomic studies provide the data required for such models.

Indeed, Bonilauri B et al. performed an in-depth proteomic analysis of human ADSCs using high-resolution mass spectrometry, identifying protein markers associated with metabolic state, stemness, and differentiation biases. Their dataset provides a robust basis for ML-driven biomarker discovery, enabling computational identification of proteins predicting ADSC quality and batch-to-batch variability [89].

Proteomics is particularly valuable for developing ML models that can classify ADSC preparations by functional potency or by their suitability for specific therapeutic indications (e.g., wound healing, osteogenesis).

### 7.3. Metabolomics + AI

Metabolomic signatures provide powerful predictors of ADSC lineage commitment and regenerative potential. Recent studies have begun integrating metabolomics with ML to classify differentiation status and detect early metabolic shifts.

A notable recent study applied kernel-based ML on ^1H–^1H TOCSY NMR data from human adipose-tissue-derived MSCs undergoing adipogenic and osteogenic differentiation. The ML models achieved high accuracy in distinguishing differentiation states and identified key metabolites associated with lineage commitment. These metabolic biomarkers can be used to predict differentiation efficiency and optimize induction protocols [85]. Finally, this integration demonstrates how metabolomics + ML can serve as an early, non-invasive readout of ADSC potency and differentiation trajectory.

### 7.4. Secretomics + ML for Potency Prediction

Secretome profiling of human ADSCs from multiple donors has shown that these cells secrete a broad repertoire of extracellular matrix proteins, growth factors, cytokines, and neurotrophic molecules, many of which are known to support tissue regeneration. In a comprehensive LC–MS study, Kalinina and colleagues analyzed conditioned media from ADSCs cultured under normoxic and hypoxic conditions and identified more than 600 secreted proteins; about 100 proteins were common to all donors, whereas many factors—including angiogenic and neurotrophic molecules—varied substantially between individuals. This donor-to-donor variability in secretome composition suggested the existence of distinct functional ADSC subtypes with potentially different regenerative capacities, providing a rich dataset for future machine-learning models aimed at donor classification and prediction of therapeutic potency based on secretome features [88].

Given its ability to capture the functional output of ADSCs, secretomics naturally lends itself to machine-learning applications aimed at donor and batch classification. By analyzing patterns within the spectrum of secreted cytokines, growth factors, and extracellular matrix components, ML models could distinguish donors with high versus low therapeutic potency, identify ADSC populations characterized by particularly strong angiogenic or immunomodulatory activity, and even predict which culture batches are most likely to meet the stringent requirements for GMP-grade manufacturing.

### 7.5. ML Applications: Donor Classification and Biomarker Discovery

Multi-omics machine-learning models can integrate transcriptomic, proteomic, metabolomic and secretomic datasets to generate predictive insights across several dimensions of ADSC biology. For example, datasets derived from single-cell transcriptomics and proteogenomics provide a foundation for models capable of anticipating key therapeutic properties such as immunomodulatory potency, angiogenic capacity and overall differentiation efficiency [87,89].

Beyond prediction, multi-omics information is increasingly used to identify biomarkers that characterize high-quality ADSC batches. Gene-regulatory patterns, metabolic fingerprints and cytokine-secretion signatures have been associated with stemness stability, proliferative potential and consistent differentiation outcomes, supporting their use as indicators of functional heterogeneity and batch potency [90,91,92].

Multi-omics ML approaches also enable the classification of ADSC donors according to their regenerative potential. Single-cell transcriptomic and proteomic datasets make it possible to distinguish donors enriched in high-stemness and highly multipotent ADSC subpopulations from those more prone to early senescence or associated with reduced therapeutic efficacy, providing an essential tool for overcoming inter-donor variability in clinical manufacturing [23,87,93].

Overall, the application of artificial intelligence to multi-omics datasets is emerging as a central pillar of ADSC research and manufacturing. By integrating transcriptomic, proteomic, metabolomic and secretomic data into unified computational frameworks, these models can predict therapeutic potency before differentiation, identify robust biomarkers of high-quality ADSC batches, stratify donors according to regenerative performance and support the standardization of GMP-compliant ADSC products. Recent integrative analyses highlight how such AI-driven, systems-biology approaches represent one of the most powerful strategies to accelerate ADSC translation toward clinical applications [90,94].

### 7.6. Multi-Omics Integration as a Source of Robust and Repeatable Potency Features

While individual omics layers provide valuable insights into ADSC biology, the integration of transcriptomics, proteomics, metabolomics, and secretomics through machine-learning frameworks enables the identification of potency features that are more robust and reproducible than single-modality biomarkers. Biological systems are inherently noisy and donor-dependent; signals detected at one molecular layer may reflect transient or context-specific changes. However, when concordant patterns emerge across multiple omics levels—such as transcriptional activation of angiogenic pathways accompanied by consistent secretome enrichment in VEGF-related proteins and corresponding metabolic shifts—these convergent signatures are more likely to represent stable functional states.

Machine-learning models facilitate this integration by identifying features that consistently contribute to predictive performance across cross-validation folds, independent donor subsets, and batch conditions. Feature-selection techniques, regularization methods (e.g., ElasticNet), and ensemble models (e.g., gradient boosting) help reduce overfitting and isolate molecular variables that remain predictive across heterogeneous datasets. In this context, robustness is achieved not merely through predictive accuracy, but through stability of feature importance and reproducibility in external validation cohorts.

Importantly, multi-omics ML models can transform high-dimensional molecular data into quantitative potency scores, linking biological signatures to functional assays such as immunomodulatory activity, angiogenic capacity, or differentiation efficiency. When these signatures are validated across donors and manufacturing batches, they may serve as candidate critical quality attributes (CQAs) for GMP-compliant ADSC products.

Thus, the combination of multi-omics integration and machine learning does not simply improve prediction performance; it enables the extraction of biologically grounded, repeatable potency features that can support donor stratification, batch release decisions, and process standardization.

## 8. AI for Manufacturing and Clinical Translation

Translating ADSC-based therapies from bench to clinic requires robust, reproducible, and scalable manufacturing processes compliant with GMP standards. AI (machine learning, predictive algorithms, automation) can play a key role in: (1) standardizing culture/expansion processes; (2) enabling AI-assisted automation (e.g., in bioreactors); (3) implementing predictive batch release testing and quality control; (4) facilitating regulatory compliance, documentation, and risk assessment.

### 8.1. Standardization and AI-Assisted Bioprocess Automation

One of the core challenges in cell therapy manufacturing is variability between batches and donors. AI-based monitoring systems—integrating real-time imaging, sensor data, and predictive analytics—can help standardize processes. For instance, in the broader biopharmaceutical field, AI and ML have been applied to optimize fed-batch bioreactor processes, adjusting feeding, process parameters and monitoring viability to maximize yield while reducing manual interventions [77].

While Khaleghi et al. focused on microbial or production cell lines rather than ADSCs, their methodological framework—combining metabolic modeling, sensor readouts, and ML—is directly transferable to stem-cell bioprocessing. In theory, a similar platform could be developed for ADSC expansion bioreactors (e.g., for suspension or microcarrier culture), enabling automated control of nutrient feeds, oxygenation, pH, and cell density to maintain consistent quality.

### 8.2. Predictive Batch Release Testing and Quality Control

Beyond real-time control, AI can assist in batch release decisions by combining multi-parameter datasets (culture history, sensor logs, metabolic readouts, secretome, morphological data) to predict final cell product quality—potency, viability, differentiation potential, safety. For example, ML models have been used in other fields to forecast final product attributes from early process data, reducing reliance on end-point assays [77].

Applied to ADSC manufacturing, this approach would involve building predictive models from retrospective datasets, linking culture conditions and early in-process measurements to final potency outcomes. After appropriate validation, such tools could support faster and more consistent batch release decisions, providing an objective framework for identifying products that meet predefined quality criteria.

### 8.3. AI-Integrated Quality Framework for ADSC Manufacturing

To improve conceptual clarity and better align artificial intelligence (AI) applications with Good Manufacturing Practice (GMP)–oriented decision-making, the relationships between critical quality attributes (CQAs), potency assays, process quality standards (PQSs/PQLSs), and batch release decisions can be framed within a unified and structured perspective, as summarized in Table 5.

In ADSC manufacturing, CQAs represent the measurable biological and functional properties that define product quality and therapeutic suitability. These typically include parameters such as cell viability, sterility, immunomodulatory potency, differentiation capacity, genetic stability, and senescence burden. Each CQA is evaluated through specific assays or quantitative readouts, including flow cytometry marker panels, morphology-based imaging metrics, secretome or cytokine profiling, metabolic signatures, and functional assays such as T-cell proliferation inhibition. The results of these assays are interpreted against predefined acceptance criteria established through regulatory guidelines, internal validation studies, and historical manufacturing data.

Within this framework, AI models can operate as an additional analytical and integrative layer. Rather than replacing conventional quality-control assays or regulatory thresholds, AI systems support the interpretation of complex and high-dimensional datasets. For example, machine-learning algorithms can extract quantitative features from imaging data, integrate multi-omics profiles to predict potency outcomes, detect early deviations from expected growth or differentiation trajectories, and stratify donor or batch variability based on multidimensional signatures. In this sense, AI functions as a decision-support tool that synthesizes heterogeneous data streams into probabilistic assessments of whether predefined CQAs are likely to be met.

From a process perspective, the quality workflow can therefore be conceptualized as a continuous chain linking defined CQAs to their corresponding analytical readouts, followed by evaluation against acceptance criteria, integration of AI-based predictive or anomaly detection models, and ultimately a manufacturing decision. This decision may involve product release, temporary hold for further investigation, or rejection of the batch, depending on the convergence between measured parameters, model predictions, and predefined quality thresholds. By explicitly articulating these relationships, the role of AI becomes clearer and more consistent with regulatory expectations. AI does not supersede human oversight or established GMP criteria; rather, it enhances reproducibility, supports early risk identification, reduces subjective interpretation of complex datasets, and provides a structured basis for objective and data-driven batch release decisions in ADSC manufacturing.

### 8.4. AI for Regulatory Compliance, Documentation Automation and Risk Assessment

Regulatory compliance in cellular therapy manufacturing requires meticulous documentation of every step in the process, including donor selection, cell isolation, expansion, quality testing, batch release criteria, and full product traceability. Artificial intelligence can play an important role in supporting these requirements. Automated data-logging systems, for instance, can continuously capture and organize information from bioreactor sensors and imaging platforms, generating complete audit trails without relying on manual entry. In parallel, natural language processing tools and AI-assisted document-generation systems can streamline the creation of batch records, quality-control reports, and regulatory submissions, reducing administrative burden while improving consistency and accuracy. AI-based predictive risk-assessment algorithms can further enhance compliance by identifying deviations from expected culture behavior—such as abnormal growth kinetics, unexpected sensor values, or early signs of contamination—based on patterns learned from historical data. Although published examples of AI applied specifically to ADSC GMP manufacturing remain limited, the rapid adoption of similar technologies in biopharmaceutical production indicates that integration of AI into regulatory workflows for ADSCs is both feasible and likely to become increasingly common [95,96].

### 8.5. Robustness of AI Models Under GMP Constraints

For AI systems to be effectively integrated into GMP-compliant ADSC manufacturing, robustness under real-world production conditions is not optional but essential. Unlike controlled research environments, GMP facilities operate within tightly regulated frameworks that require full traceability, comprehensive documentation, auditability, and validated processes. In this context, AI-assisted decision-support tools—especially those contributing to batch release decisions, potency assessment, or quality control—must demonstrate stable and reproducible performance despite donor-to-donor variability, batch heterogeneity, and routine operational fluctuations.

Ensuring such robustness requires more than high predictive accuracy in retrospective datasets. Data acquisition procedures must be standardized across platforms and operators to reduce variability at the source. Models should be implemented within structured version-control systems, accompanied by continuous performance monitoring to detect drift over time. Clear documentation of training datasets, preprocessing pipelines, and model updates is equally critical, particularly when modifications occur after initial validation. Moreover, predefined performance thresholds should be established to determine whether a model remains suitable for clinical use under evolving manufacturing conditions.

Interpretability represents an additional and often decisive factor for regulatory acceptance. Black-box predictions are unlikely to be sufficient in environments where risk assessment and accountability are mandatory. Explainable AI strategies—such as feature-importance analysis, SHAP-based interpretation, or attention-map visualization—can provide insight into the biological variables influencing model outputs, thereby enhancing transparency and facilitating dialogue with regulatory authorities.

Ultimately, AI systems intended for ADSC bioprocessing must be evaluated not solely on their predictive performance, but on their stability over time, reproducibility across sites, and compatibility with GMP operational and regulatory requirements. Only through such rigorous validation can AI transition from being an experimental support tool to a reliable component of clinical-grade manufacturing workflows.

### 8.6. Regulatory Qualification Considerations for AI Tools

The regulatory expectations for AI tools in ADSC manufacturing depend strongly on their intended role within the GMP workflow. A clear distinction should be made between AI systems used solely for monitoring or decision support and those directly influencing batch release decisions.

AI tools used for process monitoring—such as anomaly detection in imaging data or predictive modeling of growth kinetics—are generally considered supportive technologies. In such cases, regulatory expectations typically include documented model training procedures, performance validation on independent datasets, data integrity assurance, version control, and clear human oversight in final decision-making. These systems are expected to demonstrate robustness, reproducibility, and traceability, but do not independently determine product disposition.

In contrast, AI tools influencing batch release decisions are considered higher-risk applications and would require substantially more rigorous qualification. Expected elements include prospective validation under GMP conditions, predefined performance metrics (sensitivity, specificity, false-positive/negative rates), clearly defined acceptance thresholds, change-control procedures for model updates, comprehensive audit trails, explainability or interpretability mechanisms, cybersecurity safeguards, and documented governance for model re-training. In such cases, the AI system becomes part of the validated manufacturing process and must meet standards comparable to other critical analytical methods.

Distinguishing these two categories clarifies both implementation pathways and regulatory burden. While AI-based monitoring tools may be introduced incrementally with appropriate oversight, AI systems used for batch release decisions would require formal validation strategies aligned with GMP principles and risk-management frameworks.

### 8.7. Limitations and Challenges

Several challenges still limit the widespread implementation of AI in ADSC bioprocessing. One of the major obstacles is the scarcity of publicly available datasets: unlike microbial systems or standardized protein production cell lines, very few research groups release detailed ADSC bioprocess datasets that include sensor logs, culture metadata or potency outcomes, all of which are essential for robust model training. Regulatory considerations add another layer of complexity, as the validation of AI-driven decision-support tools—particularly those influencing batch release criteria—may require extensive prospective datasets and tightly controlled studies. Biological heterogeneity also remains a significant limitation. ADSCs vary considerably from donor to donor, and even batch-to-batch within the same donor, making it difficult for models to generalize without large, diverse training cohorts. Finally, the quality and standardization of input data represent a fundamental bottleneck: sensor outputs, imaging data and multi-omics measurements must be harmonized across platforms and laboratories to ensure reproducibility and reliability for machine-learning applications.

## 9. Challenges and Limitations in Applying AI to ADSC Research and Manufacturing

Although AI has shown considerable potential for improving ADSC isolation, culture monitoring, multi-omics integration, and manufacturing (as described in the previous chapters), several technical and translational challenges remain.

### 9.1. Limited Availability of Large, High-Quality ADSC Datasets

AI models designed for tasks such as cell-morphology analysis, secretome profiling, single-cell transcriptomic interpretation and differentiation prediction depend on large, diverse and standardized datasets to achieve robust performance. In the case of ADSCs, however, available datasets are generally limited in size, heavily influenced by donor-specific biological variability, and generated under heterogeneous conditions, including differences in imaging platforms, media formulations and passage numbers. Moreover, these datasets are rarely made publicly accessible, further restricting the development and validation of reliable AI models in this field [97,98].

This impedes the development of robust ML/DL models for predicting ADSC yield, potency, or differentiation outcomes.

### 9.2. Data Heterogeneity Between Laboratories

As discussed in earlier chapters, ADSC imaging datasets, culture conditions and differentiation protocols display substantial variability across laboratories. When such heterogeneous data are pooled together within machine-learning pipelines, they inevitably introduce batch effects in imaging datasets—arising from differences in microscopes, illumination settings or acquisition parameters—as well as significant variability in multi-omics signatures and marked inconsistencies in secretome profiles among donors. This heterogeneity constrains the ability of AI models to generalize across studies and significantly reduces the reproducibility of their predictions.

### 9.3. Black-Box Nature of Deep Learning Models

Deep-learning tools used for ADSC morphology classification, senescence detection, lineage prediction or multi-omics integration often function as “black boxes,” generating outputs such as potency estimates or differentiation-state predictions without providing a clear biological explanation for how those conclusions were reached. In the context of ADSC-based therapies—where stringent regulatory standards demand transparency, traceability and scientific justification—this lack of interpretability poses several challenges. It complicates the acceptance of AI-driven decisions by quality-assurance teams, makes it more difficult to troubleshoot unexpected culture deviations and ultimately creates barriers to clinical translation. For these reasons, the development and adoption of explainable AI approaches will become essential before deep-learning models can be fully integrated into GMP-compliant manufacturing workflows.

### 9.4. Validation and Reproducibility Issues

Many of the AI applications described in earlier chapters—such as deep-learning models for confluency estimation, machine-learning tools for yield prediction and multi-omics frameworks for potency modeling—are typically developed and validated using small, single-center cohorts generated under narrowly defined imaging conditions and derived from a limited pool of ADSC donors. As a result, these models seldom undergo multi-center external validation, which is essential for demonstrating robustness across different laboratories, instruments and culture protocols. Without rigorous cross-laboratory testing and confirmation that performance is maintained beyond the original training environment, AI systems cannot be reliably adopted for GMP-grade ADSC manufacturing or for supporting critical decisions such as batch release.

### 9.5. Ethical and Regulatory Concerns

The integration of AI into ADSC workflows—whether for donor selection, potency prediction or automated quality control—raises several important ethical and regulatory considerations. Data privacy is a central concern, as donor medical information is increasingly linked to high-dimensional omics profiles, creating sensitive datasets that require strict protection. Questions of bias and fairness also emerge, since machine-learning models trained on limited or unbalanced cohorts may inadvertently favor certain donor groups over others. In addition, the use of automated decision-making systems introduces new challenges: for example, an algorithm might recommend rejecting a batch or classifying a donor as unsuitable, and it remains unclear how such decisions should be governed. Closely related is the issue of responsibility when an AI-generated recommendation contributes to a manufacturing error or, ultimately, a clinical failure. Addressing these concerns in parallel with evolving regulatory frameworks will be essential before AI can be safely and reliably incorporated into ADSC manufacturing pipelines.

Future research should prioritize multi-center, externally validated AI frameworks specifically designed for ADSC manufacturing, with harmonized data standards and prospective GMP-based evaluation, in order to bridge the gap between experimental performance and clinical-grade implementation.

### 9.6. Interpretability and Regulatory Recognition of AI Models

In the context of clinical translation and GMP-compliant ADSC manufacturing, interpretability of artificial intelligence models is a central requirement for regulatory recognition. While complex models such as deep neural networks may achieve high predictive accuracy, their adoption in clinical-grade workflows is unlikely without transparent and traceable decision-making mechanisms. Regulatory authorities require not only validated performance but also a clear understanding of the variables influencing model outputs, particularly when AI systems contribute to quality control, potency assessment, or batch release decisions.

Explainable AI (XAI) approaches can help bridge this gap. Feature-importance ranking in ensemble models, SHAP (Shapley Additive Explanations) value analysis, and attention-based visualization techniques provide quantitative insight into the contribution of individual molecular, imaging, or process-related features to the final prediction. In ADSC manufacturing, such tools may allow identification of biologically meaningful drivers of potency—such as specific transcriptomic signatures, secretome components, or morphological parameters—thereby linking computational outputs to established biological mechanisms.

Importantly, interpretability enhances auditability and risk assessment within GMP environments. Transparent models facilitate documentation, reproducibility checks, and regulatory dialogue, reducing concerns related to “black-box” decision systems. Therefore, in the development of AI frameworks for ADSC bioprocessing, explainability should be considered not as an optional refinement but as a design principle aligned with clinical safety, regulatory compliance, and long-term translational adoption.

## 10. Future Directions for AI-Driven ADSC Research and Translation

Building upon the advances and challenges discussed in previous chapters, several emerging technologies are expected to transform ADSC biology, bioprocessing, and therapeutic application.

### 10.1. Real-Time AI-Driven Closed-Loop ADSC Culture Systems

As discussed in Chapters 4 and 5, AI already supports non-invasive monitoring of ADSC morphology, senescence and growth dynamics. Building on these advances, a natural progression is the development of closed-loop bioreactor systems in which AI continuously interprets imaging streams and sensor data, predicts future culture trajectories—such as the approach toward senescence—and automatically adjusts key environmental parameters, including oxygen tension, media-feeding schedules and other culture conditions. By enabling real-time and autonomous optimization of the microenvironment, such systems have the potential to maintain consistent ADSC potency while significantly reducing operator-dependent variability.

### 10.2. Integration with ADSC-on-Chip or Organ-on-Chip Platforms

Future technological platforms are likely to integrate ADSC culture modules with microfluidic devices, perfusion-based culture systems and AI-driven imaging or omics analytics tools. By combining these elements into unified experimental setups, it would become possible to perform functional potency assessments—such as evaluating angiogenic or immunomodulatory activity—prior to clinical application. At the same time, these advanced phenotyping systems would generate high-quality, standardized datasets ideally suited for machine-learning workflows, thereby supporting more accurate donor classification and enabling batch-specific predictions of therapeutic potency.

### 10.3. Foundation Models for ADSC Biology

Building on the multi-omics integration described in Chapter 7, it is conceivable that large foundation models—analogous to GPT-style architectures but trained entirely on biological data—will eventually learn from vast collections of ADSC-related information, including thousands of imaging samples, comprehensive transcriptomic, proteomic and secretomic datasets, detailed culture histories and documented differentiation outcomes. By capturing patterns across these heterogeneous data sources, such models could infer ADSC potency from minimal input, simulate differentiation trajectories, optimize culture parameters in silico and even guide experimental design for ADSC-based therapies. This type of integrative, data-driven intelligence has the potential to transform ADSC research and manufacturing by providing predictive and mechanistic insights that extend far beyond what current models can achieve.

### 10.4. Federated Learning for Multi-Center ADSC Data Integration

To address the data-scarcity and heterogeneity challenges outlined in Chapter 9, federated learning approaches offer a promising solution. By enabling hospitals and research laboratories to collaboratively train shared machine-learning models without the need to exchange raw patient or culture data, these systems promote multi-center standardization while preserving donor privacy. In turn, this framework would allow ADSC datasets generated under different conditions to be integrated securely, resulting in more robust potency-prediction models and a substantial reduction in donor-specific bias. Federated learning therefore represents an important step toward building reliable, generalizable AI tools for ADSC manufacturing and clinical translation.

### 10.5. AI-Guided Personalized ADSC Therapy

By integrating clinical metadata with donor genomics, transcriptomic profiles and secretome signatures, as outlined in Chapter 7, AI systems may soon be able to predict which donor is most suitable for a particular patient, which ADSC batch possesses the strongest immunomodulatory or angiogenic properties, and which differentiation protocol is most likely to generate the desired phenotype. Such models could even inform the selection of therapeutic regimens that maximize clinical response. Taken together, these capabilities would shift ADSC-based interventions away from a generic “one-size-fits-all” strategy and toward a true precision regenerative medicine framework, where treatments are tailored to both the biological characteristics of the donor cells and the specific needs of the recipient.

### 10.6. Positioning ADSC-Oriented AI Within the Broader Landscape of AI-Driven Biomedical Discovery

Recent advances in artificial intelligence–driven biomedical research further highlight the transformative potential of integrative machine learning frameworks across diverse domains of drug development and clinical prediction. For example, Lu et al. demonstrated how advanced ML and graph-based architectures can enable clinically actionable drug–drug interaction prediction, bridging computational discovery with real-world pharmacological application [99].

Similarly, Wang et al. employed integrative multi-omics clustering combined with ensemble machine-learning strategies to define robust molecular subtypes and construct reproducible prognostic signatures in hepatocellular carcinoma, emphasizing the value of consensus modeling across heterogeneous datasets [100].

In parallel, Chen et al. illustrated how graph neural networks can mechanistically model drug–target interactions by leveraging graph-structured biological data, enabling more interpretable and biologically grounded target discovery pipelines [101].

Complementing these approaches, Song et al. developed a machine learning–derived prognostic signature through large-scale multi-cohort integration and algorithmic consensus modeling, underscoring the importance of stability, cross-validation, and clinical generalizability in predictive frameworks [102].

Collectively, these representative studies demonstrate how multi-omics integration, graph-based modeling, ensemble learning, and rigorous cross-cohort validation can generate robust molecular subtypes, prognostic models, and clinically oriented predictive systems. Within this broader AI-driven biomedical ecosystem, the application of similar integrative and validation-oriented strategies to ADSC manufacturing and potency prediction represents a natural and necessary progression toward standardized, regulatory-aligned, and clinically translatable cell-therapy platforms.

## 11. Conclusions

AI is rapidly reshaping how ADSCs are studied, cultured, and prepared for clinical use. Across the workflow—from donor selection and tissue pre-screening, through isolation and expansion, to differentiation, multi-omics characterization, and manufacturing—AI provides tools to extract quantitative information that was previously inaccessible or too labor-intensive to obtain. The sections of this review highlight how machine learning and deep learning already support image-based monitoring of ADSC morphology and senescence, optimization of culture media and process parameters, and interpretation of complex transcriptomic, proteomic, metabolomic, and secretomic datasets.

A consistent theme emerging from the literature is that AI excels at linking early, minimally invasive measurements to later functional outcomes. Morphology-based models can predict expansion kinetics and immunomodulatory potency. Metabolomics and secretomics combined with ML can identify signatures associated with differentiation success or regenerative efficacy. Single-cell transcriptomics enables the discovery of ADSC subpopulations with superior stemness or lineage bias. These capabilities make AI a powerful ally for donor stratification, batch release decisions, and rational design of differentiation protocols.

At the same time, the review underscores that many current applications are still proof-of-concepts and often developed on related mesenchymal stromal cell systems rather than ADSCs alone. The field is constrained by limited, heterogeneous datasets and by the black-box nature of many deep learning approaches, which complicates regulatory acceptance. Robust external validation, transparent model interpretation, and harmonization of data acquisition across laboratories will be essential before AI tools can be embedded into GMP-grade ADSC manufacturing pipelines.

Looking ahead, several converging trends are likely to accelerate progress: real-time, closed-loop culture systems driven by AI; the integration of ADSC cultures with organ-on-chip platforms for functional potency testing; foundation models trained on large multi-modal cell datasets; and federated learning frameworks enabling multi-center model training without compromising data privacy. Together, these advances point toward a future in which ADSC-based therapies are supported by data-driven, highly standardized, and increasingly personalized manufacturing workflows.

In conclusion, AI should not be viewed as a replacement for biological insight, but as an enabling technology that can systematically capture and exploit the complexity of ADSC biology. When combined with rigorous experimental design, high-quality data generation, and appropriate ethical and regulatory oversight, AI has the potential to significantly enhance the safety, reproducibility, and therapeutic impact of ADSC-based regenerative medicine.

## Figures and Tables

**Table 1:** Overview of artificial intelligence (AI) applications across the entire adipose-derived stem/stromal cell (ADSC) pipeline. The table summarizes the main AI techniques, objectives, and advantages at each stage, highlighting current maturity levels of evidence for ADSC-specific and MSC-related systems.

ADSC Workflow Stage	AI Techniques	Primary Objectives	Key Advantages	Evidence Level
Donor selection & tissue pre-screening	Random Forest, Regression, Deep Learning on histological images	Predict ADSC yield, viability, inflammation status	Reduces donor-dependent variability	Medium (MSC + adipose tissue studies)
Isolation & SVF processing	Bayesian optimization, Active learning	Optimize digestion time, enzyme concentration, centrifugation	Higher SVF yield and viability	Low–medium (non-ADSC systems)
Expansion & culture monitoring	CNNs, U-Net, RNN/LSTM, Self-supervised DL	Track morphology, confluency, proliferation, senescence	Non-invasive, real-time, standardized monitoring	High (MSC + ADSC imaging data)
Quality control (QC)	CNNs, Virtual staining, ML classifiers	Detect senescence, contamination, deviation from healthy phenotypes	GMP-oriented automated QC	Medium–high
Differentiation	Deep learning, Clustering, Multi-omics ML	Predict differentiation state, optimize induction protocols	Faster optimization, reduced reagent use	Medium
Multi-omics potency prediction	Feature importance, gradient boosting, SHAP, integrative ML	Predict immunomodulatory and angiogenic potency	Enables donor and batch stratification	Medium–high
GMP bioprocessing	Predictive modeling, RL, anomaly detection	Automated control of bioreactors and batch release	Higher scalability, reduced lot variability	Low–medium

**Table 2:** Key AI terms and definitions. Summary of artificial intelligence (AI) approaches—including machine learning (ML) and deep learning (DL)—and their main applications in ADSC culture monitoring and process standardization.

Term	Short Definition	Example in ADSC Manufacturing/QC
Artificial Intelligence (AI)	Broad set of computational methods enabling automated decision-making and pattern recognition from data.	End-to-end support for process monitoring, quality control, and standardization across the ADSC workflow.
Machine Learning (ML)	Subfield of AI in which models learn predictive or classification rules directly from data rather than being explicitly programmed.	Predicting cell yield/viability from donor metadata (age, BMI, comorbidities) or culture conditions.
Deep	Subfield of ML based on multi-layer neural networks, particularly effective for high-dimensional data such as images.	CNN-based analysis of label-free microscopy to infer confluency, morphology, or differentiation state.
Supervised learning	ML paradigm trained on labeled examples (input → known output) to perform classification or regression.	Classifying “senescent vs. non-senescent” cells or predicting potency scores from imaging/omics features.
Unsupervised learning	ML paradigm that identifies intrinsic structure in unlabeled data (e.g., clustering, dimensionality reduction).	Detecting subpopulations in scRNA-seq datasets or clustering morphological phenotypes to quantify heterogeneity.
Computer vision	Methods to extract quantitative information from images, often powered by DL.	Automated cell segmentation/counting, growth tracking, and detection of abnormal culture patterns in real time.
Generative AI (e.g., GANs)	Models that generate realistic synthetic data or enhance data quality (e.g., denoising, super-resolution).	Data augmentation for training robust image models or improving microscopy image quality for QC pipelines.
Predictive modeling & optimization	Data-driven strategies to forecast outcomes and optimize process parameters using iterative learning.	Optimizing media formulations and culture parameters; early prediction of batch performance and reproducibility.

**Table 3:** AI Techniques and their ADSC-specific applications. Summary of artificial intelligence methodologies applied to ADSC biology and manufacturing. The table outlines the type of algorithm, data sources, specific applications, and main limitations.

AI Technique	Category	Data Input	Applications in ADSCs	Current Limitations
CNN/U-Net	Deep Learning	Bright-field, phase-contrast images	Cell segmentation, confluency estimation, morphology assessment, senescence detection	Requires annotated datasets; variability across imaging systems
Self-supervised DL	Representation Learning	Unlabeled time-lapse data	Morphodynamic profiling, state transitions, trajectory prediction	Limited interpretability
Random Forest/Gradient Boosting	Machine Learning	Donor metadata, omics, morphology	Predict yield, potency, expansion rate	May underperform compared to DL
SVM/Unsupervised clustering	Classical ML	Proteomics, metabolomics, Raman/FTIR	Differentiation stage classification, donor stratification	Sensitive to batch effects and normalization
RNN/LSTM	Deep Learning	Time-series imaging or sensor data	Proliferation dynamics, differentiation kinetics	Requires large temporal datasets
GANs	Generative DL	Low-resolution images	Super-resolution, data augmentation	Hard to validate in GMP settings
Bayesian Optimization	Active Learning	Experimental parameters	Optimization of media composition, enzymatic digestion	Limited ADSC-specific implementations
Reinforcement Learning	Decision-making AI	Sensor inputs from bioreactors	Automated control of cultures (feeding, oxygen, perfusion)	Still conceptual for ADSCs
Integrative Multi-Omics ML	Multi-modal ML	RNA-seq, proteomics, secretomics, metabolomics	Biomarker discovery, potency prediction	Scarcity of large multi-omics ADSC datasets

**Table 4:** **Key literature on AI applied to ADSCs and MSCs.** Representative studies applying artificial intelligence methods to ADSCs or closely related mesenchymal stromal cell systems. The table summarizes cell type, AI technique, data modality, objectives, and main findings.

Study	Cell Type	AI Technique	Data Type	Objective	Main Findings
Imai et al. (2022) [78]	BM-MSC + ADSC	Morphology-based ML	Time-lapse images	Early prediction of growth and immunosuppressive potency	>95% accuracy in “risky” donor lot detection
Mota et al. (2021) [63]	MSC	Segmentation + ML classifiers	Phase-contrast images	Automated phenotype classification	High sensitivity and specificity
Kusumoto et al. (2021) [64]	ADSC	CNN	Morphological features	Senescence detection and scoring	Introduced “senescence score” for QC
Brooks et al. (2021) [83]	iPSC/MSC	CNN	Live-cell imaging	Quantifying adipogenic differentiation kinetics	Captured donor-specific differentiation dynamics
Augustyniak et al. (2024) [84]	ADSC	Supervised ML	FTIR + Raman spectroscopy	Identify stages of adipogenesis	Metabolic spectral markers predict differentiation
Migdadi et al. (2023) [85]	ADSC	Kernel ML	NMR spectra	Track metabolic shifts during differentiation	Accurate discrimination of adipogenic vs. osteogenic states
Lin et al. (2023) [80]	ADSC	ML feature importance	scRNA-seq + CellTagging	Reconstruct osteogenic trajectories	Identified regulators (SERPINE2, SFRP1, etc.)
Kalinina et al. (2015) [88]	ADSC	Clustering/statistical profiling	Secretome (LC-MS)	Identify functionally distinct ADSC subtypes	Strong donor-dependent secretome variability
Asmar et al. (2024) [70]	iPSC	DL (virtual staining)	Bright-field images	Predict nuclear morphology from unlabeled images	Applicable for label-free QC
Pylvänäinen et al. (2023) [72]	Stem cells	DL imaging review	Multi-modal imaging	Standardizing live-cell analytics	Conceptual foundation for ADSC imaging QC

**Table 5:** **AI-Integrated quality decision framework for GMP-oriented ADSC manufacturing.** Schematic representation of the structured relationship between critical quality attributes (CQAs), corresponding analytical assays or quantitative readouts, predefined acceptance criteria, the role of artificial intelligence (AI) models (e.g., feature extraction, predictive modeling, anomaly detection, risk stratification), and the resulting manufacturing decision (release, hold, or further investigation). The framework illustrates how AI functions as a decision-support layer within regulatory-defined quality control processes, enhancing reproducibility and risk assessment without replacing established GMP acceptance thresholds.

CQA	Assay/Readout	Acceptance Criteria	AI Role	Decision Impact
Viability	Trypan blue, automated imaging	≥85% viable cells	Image-based ML classifier	Release
Immunomodulatory potency	T-cell inhibition assay	≥predefined inhibition %	Predictive regression model	Release
Senescence burden	Morphology CNN score	Below threshold	Senescence detection CNN	Investigate
Sterility/contamination	Sensor + imaging	No anomaly detected	Anomaly detection model	Hold
Differentiation capacity	Gene expression panel	Marker expression range	Multi-omics classifier	Release

## Data Availability

No new data were created or analyzed in this study. Data sharing is not applicable to this article.

## Abbreviations

The following abbreviations are used in this manuscript:

ADSC	Adipose-derived stem cell
AI	Directory of Open Access Journals
ML	Machine learning
DL	Deep learning
QC	Quality control
GMP	Good Manufacturing Practice
BMI	Body mass index
SASP	Inflammatory secretory profile
CQAs	Critical quality attributes
PQSs	Processing quantitative standards
PQLSs	Processing qualitative standards
CQAa	CQAs
SVMs	Support vector machines
CNNs	Convolutional neural networks
RNNs	Recurrent neural networks
GANs	Generative adversarial networks
RL	Reinforcement learning
